# Progression of Notch signaling regulation of B cells under radiation exposure

**DOI:** 10.3389/fimmu.2024.1339977

**Published:** 2024-03-08

**Authors:** Xin Shu, Jie Wang, Huihong Zeng, Lijian Shao

**Affiliations:** ^1^ Department of Occupational Health and Toxicology, School of Public Health, Jiangxi Medical College, Nanchang University, Nanchang, China; ^2^ Jiangxi Provincial Key Laboratory of Preventive Medicine, Jiangxi Medical College, School of Public Health, Nanchang University, Nanchang, China; ^3^ Department of Histology and Embryology, School of Basic Medicine Sciences, Nanchang University, Nanchang, China; ^4^ Jiangxi Provincial Key Laboratory of Interdisciplinary Science, Nanchang University, Nanchang, China

**Keywords:** radiation, B cells, Notch signaling, spleen, HSC

## Abstract

With the continuous development of nuclear technology, the radiation exposure caused by radiation therapy is a serious health hazard. It is of great significance to further develop effective radiation countermeasures. B cells easily succumb to irradiation exposure along with immunosuppressive response. The approach to ameliorate radiation-induced B cell damage is rarely studied, implying that the underlying mechanisms of B cell damage after exposure are eager to be revealed. Recent studies suggest that Notch signaling plays an important role in B cell-mediated immune response. Notch signaling is a critical regulator for B cells to maintain immune function. Although accumulating studies reported that Notch signaling contributes to the functionality of hematopoietic stem cells and T cells, its role in B cells is scarcely appreciated. Presently, we discussed the regulation of Notch signaling on B cells under radiation exposure to provide a scientific basis to prevent radiation-induced B cell damage.

## 1 Introduction

Delayed recovery of the immune system after radiotherapy (RT) is one of the main reasons for death in patients with malignant tumors. The higher the dose of ionizing radiation (IR) used in radiotherapy, the more severe the impairment of the functioning of the immune system. B cells are one of the highly sensitive cells to IR. B cells are derived from common lymphoid progenitor (CLP) which can be differentiated from hematopoietic stem and progenitor cells (HSPCs) in the bone marrow (BM). Even though Notch signaling is essential for HSPCs and lymphocyte development, it is still important to in-depth investigate how Notch signaling affects B cells injury and regeneration under irradiation. In the present review, we summarize the progression on the role of the Notch signaling pathway in regulating B cells, which may be applied to the immune system damage and recovery after ionizing radiation.

## 2 Development and radiosensitivity of B cells

**Figure 1:**
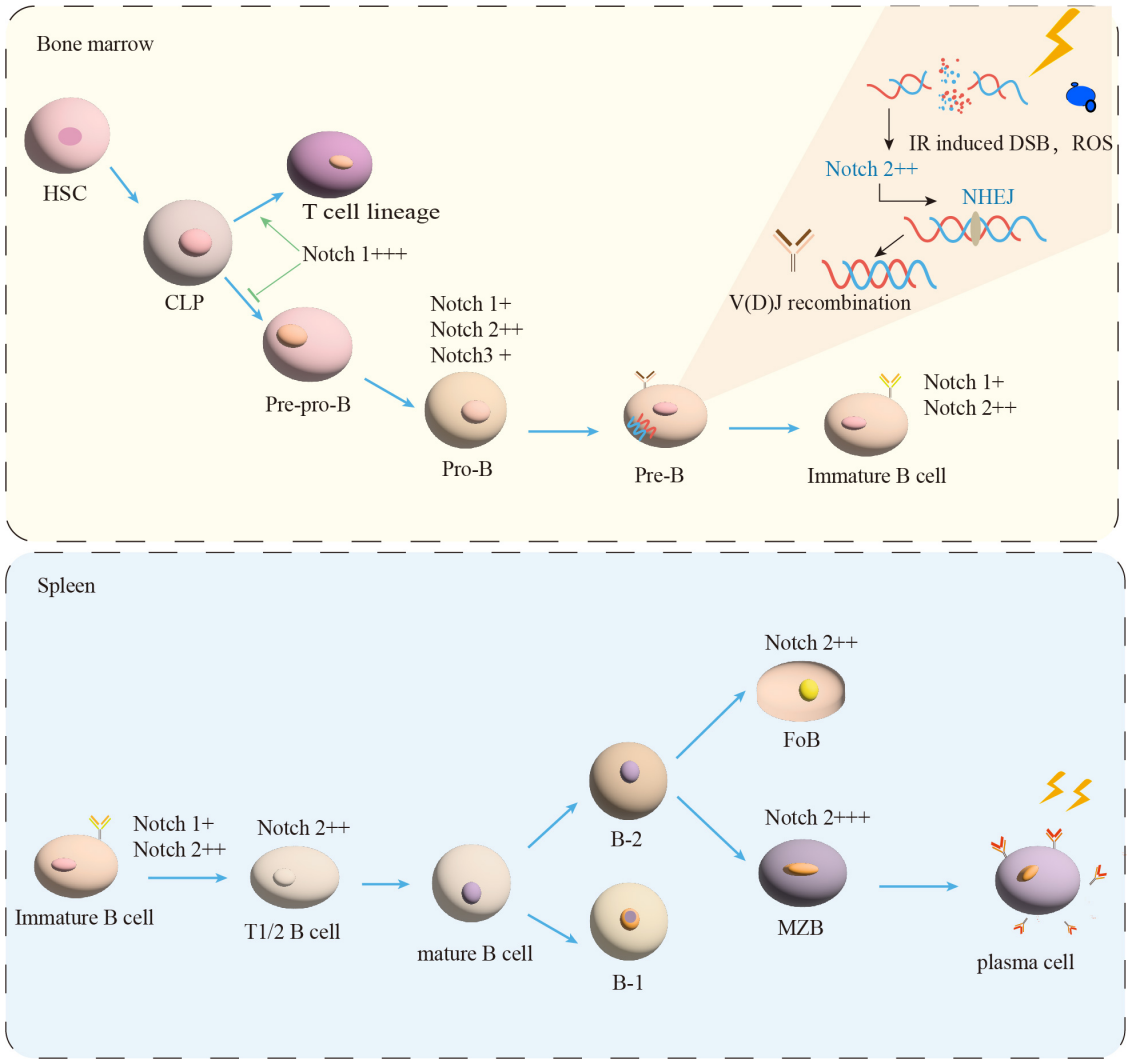
B-cell maturation and differentiation. In the BM, the B-lymphocyte lineage originates from hematopoietic stem cells (HSC) and progressively differentiates into pre-pro-B, pro-B, and pre-B cells. Immature B cells leave the BM and transfer to the spleen for further development into transitional B cells (T1/2). Mature B cells are composed of B-1 and B-2 cells. B-2 cells are classified into FoB and MZB cells. MZB cells continue to develop into plasma cells. IR induced DSB and ROS production. NHEJ is essential for the process of V(D)J recombination in Pre-B cells. Notch signal (+) expression, (++) moderated expression, and (+++) high expression.

With impressive outcomes, radiotherapy (RT) has been extensively utilized in the treatment of B-cell malignancies (1). The decrease of B cells counts in patients following radiotherapy has long been recognized as a concern. RT has the potential to have both immunostimulatory and immunosuppressive effects (2, 3). The relationship between IR and the immune system is complicated (4). The development and differentiation of B cells are regulated by genes and external factors in the spleen and BM (5). The spleen, as the largest immune organ, contains many B cells and subsets, which is essential for immune regulation (6). The process of B cell development in human is showed in **Figure 1**. In agreement with previous findings in human, HSPCs in the BM can differentiate into Preprogenitor B cells (Pre-pro-B cells), Progenitor B cells (Pro-B cells), Precursor B cells (Pre-B cells), and immature B cells expressing IgM through the rearrangement of immunoglobulin genes (7, 8), which is independent of antigenic stimulation, named antigen-independent stage (8). The immature B cells are drained from the BM to the peripheral and undergo differentiation into transitional B cells (8, 9).

Transitional B cells play a key role in linking BM immature and peripheral mature B cells (9). Human transitional B cells are subdivided into two populations: transitional B cells of type 1 (T1) and type 2 (T2) (10–12). It has been demonstrated that in the adult spleen T1 B cells develop into T2 B cells in 2 days (11–13). After passing the transitional stage, they become mature B cells (12, 14). The transitional B cells development in mice is similar to human (8, 12, 14). It is currently believed that peripherally developed mature B cells can be divided into two types of B cells: B-1 and B-2 (9, 15). B-2 B cells further differentiate into follicular B cells (FoB) and marginal zone B cells (MZB) in the human (12, 16, 17), as shown in **Figure 1**. The multiple critical phases from the BM of the central immune organ to the development of mature B cells in the peripheral immune organs are considered to be important targets for shaping the mature B cells pool (18).

How to maintain the homeostasis of sufficient B cells in the BM and peripheral spleen B cell compartments is still unclear in response to exposure to IR. It is known that lymphocytes among blood cells are the most sensitive to radiation (19–22). B cells are susceptible to radiation-induced apoptosis (4, 20, 23–25). Through determining the frequency of apoptosis in different lymphocyte subpopulations of peripheral blood mononuclear cells (PBMCs) under irradiated (24 h, 2 Gy), the following order of radiosensitivity was observed: B cells > memory T cells > NK cells (26). According to previous studies, different subpopulations of B cells have different radiosensitivities (20, 26). For example, exposed to X-radiation(0, 1, 2, 3, 4, 5 Gy), the rank order of increasing sensitivity was pre-B>pro-B>mature B cells (27). Furthermore, previous data had shown that pre-B cells were ultra-sensitive to radiation and underwent apoptosis at very low levels of radiation exposure (28–30). Moreover, the population of T1 B cells in the spleen was severely decreased 24 hours after irradiation (2, 8, 20 Gy) while the population of T2 B cells was increased (27). To determine the radioresistance of the mature B cell subsets, purified splenic B-2 cells and peritoneal B-1 cells were exposed to 2 Gy of irradiation (27, 31). B-2 cells were found to rapidly undergo apoptosis following irradiation, whereas B-1 cells maintained viability (31). A deeper analysis of the sequence of the B cell receptor (BCR) has shown that radiation induces alterations in B cells repertoire and clonogenicity (32, 33). Radiation increases the differentiation of nuclear plasma cells from tumor-antigenic B cells (34). Immunosuppression and imbalance of immune homeostasis induced by IR may lead to inflammatory responses and death in exposed organisms (35). The spleen experiences histomorphologic changes following radiation at varying doses (36). These changes include a reduction in the splenic index, a shrinkage of the B cells follicular zone, a decrease in the area of the red medulla oblongata, dense and compact splenic trabeculae, aggravation of splenic white medulla atrophy, and a massive decrease in lymphocyte counts (36, 37).

Previous studies have shown that the main target of IR is intracellular genetic materials (38), including direct damage such as double-strand breaks (DSBs), single-strand breaks (SSBs), and inter-strand crosslinks (ICLs) (39, 40). The generation of DSBs induces replicative stress that disrupts the stability of the cellular genome (41). DNA repair can be carried out through pathways such as non-homologous end joining (NHEJ), which is the main approach to repair damaged DNA in mammalian cells and occurs throughout the cell cycle (42). NHEJ is required for the repair of DNA double-strand breaks associated with the normal physiological Rag endonuclease-related process of V(D)J recombination, which is important for B-cell development (42–44) (**Figure 1**). Once DNA repair defects are created, they will affect hematopoietic and immune regulation, leading to bone marrow failure (BMF) and immune system malignancies (45). X-ray irradiation has been shown to cause an increase in the number of micronuclei in mouse spleen and bone marrow cells, which is a major damage of SSB and DSB (46, 47). Radiation may interact with free or bound water (35) in the cell to generate reactive oxygen species (ROS) (35, 48). Additionally, excessive accumulation of ROS, a byproduct of normal oxidative metabolism in eukaryotic cells is the main factor causing indirect oxidative stress (49, 50). ROS damages to B cells by interfering with the structure and function of DNA, lipids (51), and proteins (48, 52). A previous study demonstrated that the overproduction of ROS after radiation exposure resulted in the formation of apoptotic nuclei leading to cellular apoptosis, inducing neutrophil accumulation and inflammatory response (53–55). Nuclear factor erythroid-2-related factor 2 (Nrf2) as the major effector of ROS in the cell regulates Notch activation to counteract the deleterious effects of ROS, such as DNA damage and apoptosis (56, 57). Paul has reported that ROS acts as a rheostat to regulate the Nrf2-Notch pathway (56). To further confirm ROS regulation of Nrf2-Notch, relevant studies have demonstrated that the delayed repair seen in the NRF2^-/-^ airway after injury was rescued by activation of Notch (56, 58). NRF2 can expand HSPCs by activating Notch1 signaling in irradiated mice after ROS (57). In the case of oxidative stress after radiation, the large amount of ROS produced can activate the ROS-Nrf2-Notch pathway to regulate cell proliferation and thus reduce ROS level (56). To gain a better understanding of how Notch signals are involved in radiation response, correlated studies have found that knockdown of Notch1 or Notch2 increased the radiosensitivity of glioma stem cells (59). In the acute setting, radiation has previously been shown to increase endothelial Notch signaling, especially Notch1 and Notch2 (60–62), which were supported by the upregulation of the Notch pathway components *Jagged1* and *Hey1* (59, 61). Kondelaji observed that 8 Gy of irradiation in pulmonary endothelial cells increased transcription of Notch2 target genes *Hes1* and *Hey2* at 6, 24, and 72 h following irradiation (62). These results further validate the important role of the Notch pathway in the regulation of radioresistance, suggesting that Notch activation may be required for radioresistance.

In the aforementioned studies, we found that B cells and their subsets were damaged to varying degrees after radiation exposure, such as cell apoptosis. Therefore, the extensive generation of ROS and the impact of DNA damage on B cells during radiotherapy still require our attention. More studies indicated that the Notch signaling pathway is activated under conditions of oxidative stress induced by radiation (57). It is worth further investigating whether the Notch signaling pathway, a crucial regulator in B cells lineage development, plays a regulatory role in B cells after radiation injury.

## 3 Notch signaling pathway

**Figure 2:**
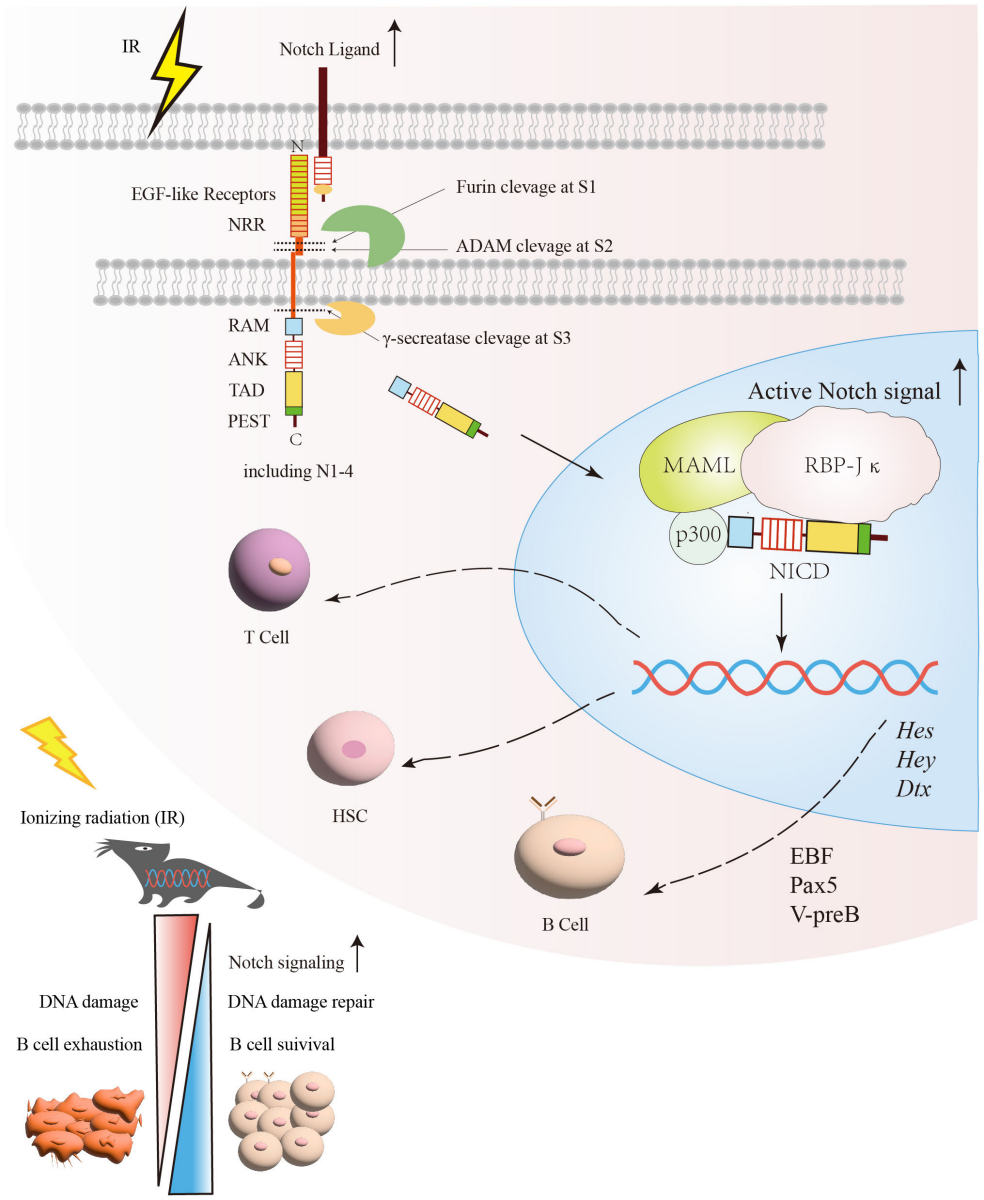
The protective effect of the Notch signaling pathway on B cells under irradiation exposure. After exposure to radiation, DNA damage and other associated harmful effects can lead to B cells exhaustion. Following radiation injury, the activation of Notch and its related signaling pathways facilitates DNA damage repair and promotes B cells survival. Subsequently, the relative enzymes are released and target specific sites (S1, S2, S3) within the Notch signaling pathway for cleavage in order to generate NICD. Once NICD enters the nucleus, it can recruit MAML and RBP-Jκ, releasing corepressors and recruiting coactivators. This process promotes the transcription of Notch target genes, such as *Hes*, *Hey*, and *Dtx*. Through the regulation of Notch signaling, it promotes the recovery of B cells after irradiation. NICD, Notch intracellular domain; RBP-Jκ, Recombination signal-binding protein for immunoglobulin kappa J region; MAMLs, Mastermind-like proteins.

The Notch signaling pathway is a G protein-coupled receptor (GPCR) and enzyme-linked receptors-mediated meristem signaling pathway controlling diverse aspects of the differentiation and maturation of lymphocytes and HSC (63, 64). As shown in **Figure 2**, the Notch signaling pathway consists of four components: the receptors, ligands, the CSL DNA-binding proteins, and downstream target genes (65). Currently, four Notch receptors are known, namely Notch1, Notch2, Notch3, and Notch4. Five ligands namely Jagged1, 2, and Delta-like ligands 1, 3 and 4 (63). Structurally, the Notch receptor is a single transmembrane heterodimer consisting of an extracellular ligand-binding domain and intracellular structural domain (66), which constitutes a transmembrane region and the intracellular part that mediates the receptor ligation signal (67, 68). The extracellular region of the Notch receptor is an elongated structure (69), the N-terminal end of the protein located outside the cell contains multiple epidermal growth factor-like receptor (EGF-like receptor) repeats (63). The numbers of EGF-like repeats vary among Notch family members (68). The EGF-like receptors are followed by the negative regulatory region (NRR) (63), which prevents premature signaling of the Notch receptor by blocking protein hydrolysis cleavage sites (70). Near the transmembrane structural region of the NRR are Furin protease cleavage site 1 (S1), a disintegrin and metalloproteinase domain (ADAM) cleavage site 2 (S2), and a γ disintegrin and metalloproteinase cleavage site 3 (S3) (71). The RBP-Jκ association module (RAM), ankyrin repeat sequence (ankyrin, ANK), transcriptional activation domain (TAD) (72), and proline/serine/threonine-rich motifs (PEST) are composed of the intracellular domain of Notch receptor (ICN) (73–75). The PEST structural domain located at the C-terminal end contributes to Notch degradation (73–75). The TAD is capable of autonomous transcriptional activity and directly binds to the coactivators PCAF and GNC5 (64, 76). Upon binding of the Notch receptor and ligand, the Notch receptor is cleaved by ADAM family proteins at site 2 (S2), followed by the cleavage of site 3 (S3) by γ-secretase (77), which ultimately releases the Notch intracellular domain (NICD), making the NICD readily localized to the nucleus, where it binds to the coactivator (Mastermind-like-1, MAML1) and the transcriptional repressor, RBP-Jκ, to promote the activation of target gene expression such as *Hes, Hey* and *Dtx* gene families (78–80).

Notch signaling regulates B cells maturation depending on RBP-Jκ (also called REPJ), the sequence-specific transcription factor, which is formed in B cells to promote B cells development under certain special conditions (81–83). Interestingly, the ADAM family is particularly important in regulating Notch signaling activation involved in lymphocyte development and maturation. It has been shown that ADAM10 is required for initiating Notch2 signaling in B cells and plays an important role in the development of the entire marginal zone B cells lineage (84–86). In ADAM10 deficient gene mice, the expression of *Dtx1* and *Hes1*, *Hes10*, and other downstream target genes of the Notch signaling pathway were significantly suppressed in T1 B and MZB (84, 85). Taken together, Notch signaling, especially Notch2, is critical for proper B cells development.

## 4 Expression of Notch receptors in B cells and its subsets

The four Notch receptors have different functions in different cells due to the specificity of their receptor structures (87). Notch1 and Notch3 are highly expressed in thymus T-cells (88). Notch4 is less expressed in mouse B cells (89–91). In particular, it was recently reported that Notch2 is mainly expressed in B-cells (92–94). In BM, Notch1 signaling inhibits the developmental differentiation of HSPCs to B cells, thereby promoting early T cells development (72, 95). The inclination of Notch1 to promote T cells development is more pronounced in the BM (96), thus prompting the inquiry into how B cells respond to Notch1 signaling to sustain their own developmental processes (97). The B-cell lineage factor Pax5 has been identified as capable of inhibiting the expression of Notch1 and attenuating the tendency of T cell differentiation, thereby ensuring the development of the B-cell lineage (98–100).

To further investigate the function of Notch on B cells development, recent studies have suggested that Notch2 is expressed throughout B cells development which is particularly highly expressed on a subpopulation of spleen-matured B cells (101, 102). Notch2-mediated RBP-Jκ signaling is essential for MZB development (83, 103, 104). The data supporting a role for Notch2 signaling in MZB were obtained using CD19-Cre knockdown of the RBJ-Jκ allele in B cells, which exhibited a significant reduction in the number of MZB and a modest increase in the number of FoB (83, 105).

The Msx2 interacting nuclear target protein (MINT) promotes FoB development by interacting with RBP-Jκ, thereby inhibiting Notch-RBP-Jκ-mediated signaling (90). MINT was found to be a negative regulator of Notch/RBP-J-dependent signaling (90), and more highly expressed in FoB than that in MZB (90, 106). MINT deficiency resulted in more efficient differentiation of splenic B cells into MZB with a concomitant decrease in FoB (92). Notch2 influences B cells lineage differentiation toward MZB and FoB by regulating the expression profile of RBP-Jκ (92). A related report found that Notch2 expression was low in B-1 cells in the spleen, but higher in B-2 cells including FoB and MZB (107–109). This is further evidence that Notch2 plays a crucial role in late developmental differentiation of B cells (103, 110, 111). Notch1 is preferentially expressed in immature T cells (112–114) while Notch2 is expressed in mature B cells (93), indicating that Notch1 and Notch2 have functionally distinct roles in the lymphocyte development (93, 115–117). This is due to their different expression patterns or specific regulation in lymphogenesis (93). The function of Notch3, due to its low expression levels in mouse B cells, is not clear during B cells development (93). The distinguishing feature of Notch4 is its reduced number of EGF repeats, absence of a transcriptional activation domain, and lack of cytokine response proteins while exhibiting robust expression in endothelial cells (118). The activation of the Notch4 signaling pathway enhances the activity of HSPCs and promotes the proliferation of immature T cells lineage, resulting in impaired B cells development. These findings indicate that Notch4 may impede the differentiation of HSPCs into B cells (119, 120).

## 5 Regulation of irradiated-B cells by Notch signaling

Since the body requires a sufficient number of lymphocytes for immune monitoring (21), it is essential to maintain a pool of primary lymphocytes at different stages (121). Radiation-induced immunosuppression leads to the emergence of opportunistic infections (121, 122). The damage caused by these infections can be fatal depending on the radiation dose, dose rate, and duration of exposure (122). Therefore, protection of immunoreactive cells from radiation-induced damage is important for immune hemostasis. Among these immune cells, B cells play a major role in the humoral immune response. Extensive studies of B cells development have helped to determine the severity of radiation damage to B cells at various stages (21). Epidemiological data showed that exposure of infants or young adult mice to IR increases the risk of precursor B-cell tumors (123).

The impact of irradiation on B cells development was assessed, revealing an augmentation in the populations of Pro-B and Pre-B cells within the BM of irradiated mice, while a substantial reduction was observed in the numbers of Pre-pro-B cells (124–126). Pre-B, as the next cell subset in the developmental stage of Pro-B, is the earliest type of cell to produce Pre-B cells signaling receptors (Pre-BCRs), which stimulates the proliferation of developing B cells (33). Interestingly, the numbers of immature B cells in BM significantly decreased on 3 days after irradiation and reversed to a significant increase after 14 days (30). The resistance to radiation exhibited by Pro-B and later developing Pre-B may be related to the differentiation stages of the cells. Differentiated cells are usually more resistant to radiation than undifferentiated cells (127). According to the aforementioned analysis, relevant studies have revealed significant alterations in the total count of B lymphocytes at various stages subsequent to total body irradiation (30, 128, 129). Further analysis on these points showed that a significant increase in immunoglobulin heavy chain rearrangements and a decrease in immunoglobulin light chains in B cells 1-2 weeks after irradiation (21, 128, 130, 131). Importantly, heavy and light chain immunoglobulin genes were recombined by V(D)J and rearranged at the Pro-B and Pre-B stages, respectively, depending on RAG-1 and RAG-2 DNA nucleic acid endonucleases (30, 132, 133). The body achieves DNA repair through NHEJ. Radiation activates the DNA damage repair response pathway, which includes the NHEJ pathway required for B-cell development (30, 133). This suggests that after irradiation 1-2 weeks, the period of transition from pro-B to pre-B cells, is a critical period for early B cell subsets to process DNA damage repair (21, 134).

Although precursor B cells are highly sensitive to radiation-induced DNA damage within 1-2 weeks (21). To attenuate radiation-induced damage to the lymphatic system (135), a subpopulation of B cells achieves rapid regeneration and differentiation under the regulation of Notch signaling (27, 136). Notch signaling is involved in the stages of early B-cell development probably through the regulation of early B-cell factor (EBF) (121, 137). *Pax5* functions to activate pre-B cell-restricted target initiation factors (e.g. Cd79a, λ5, V-preB, and B29) (137). A regulatory network consisting of the transcription factors *EBF1*, *Pax5*, *E2A*, and *Foxo1* is closely associated with B-cell gene activation and lineage formation (121). In this network, both *EBF1* and *Pax5* are involved in B cell development by repressing genes (98, 121, 137–139), which are associated with T cell lineage development (99, 121). *Pax5* represses genes encoding cell surface receptors (99), such as Notch1, while *EBF1* represses genes encoding T cell lineage-promoting transcription factors, such as TCF1 and GATA3 (82, 98–100, 121, 137). *EBF* may be a key regulator of Notch signaling in pre-B cells generation, mainly through genes encoding key components of the pre-B cells receptor (99, 121, 140, 141). In addition, 72% of the genomic binding sites in pre-B cells were found to overlap with *EBF1* binding sites (82, 121, 137). MZB expressing *EFB1* also requires Notch2 signaling for maintenance, suggesting that Notch signaling activates these transcription factors involved in B cells development (93, 121). To investigate whether different Notch ligands influence early B-cell differentiation, Delta-1 and Jagged-1 were found to have different effects on early B-cell differentiation (128). Delta-1-4 signaling prevented Pro-B cells differentiation while promoting the development of cell populations with T/Nk progenitor cell phenotypes (128). In contrast, Jagged-1 did not interfere with the development of HSPCs to B lymphocytes (128, 134). To investigate the effects of radiation on peripheral splenic B cells subsets, relevant data showed that B-regs cells, memory B cells, transitional (T1, T2) B cells, and mature B cells showed different degrees of reduction in numbers within 24 hours after irradiation, whereas plasma cells differentiated from MZB showed a high degree of resistance to radiation (8, 30, 142). BAFF signaling and NF-κB signaling are required for the development of T2 B cells into FoB, which recirculates back to secondary lymphoid organs through the bloodstream and lymphatics (30, 93). Thus, FoB tends to be more genetically diverse than MZB in terms of IgV(D) genes (93). FoB interacts with T helper (Th) cells to form germinal centers, undergo class-switch recombination (CSR) and somatic hypermutation (SHM), and ultimately produce high-affinity antibodies or memory B cells (62, 77). Moreover, Notch2 is important for the development of T2 B cells into MZB (93). MZB is located in the marginal sinus at the outer edge of the splenic follicle, which is the junction of the red and white pulp (80). MZB participates in the thymic-independent antigenic immune response, allowing for the production of large numbers of IgM-producing, short-lived plasma cells (81). In addition, ADAM10 has been shown to play a key role in Notch2-mediated MZB development. Bone marrow transplantation of irradiated mice with recombinant ADAM10 revealed that the lymph nodes of the transplanted mice had normal lymphoid structure and the MZB in the cortical area were restored to normal (143). The study demonstrated that Notch-mediated ADAM10 expression restored secondary lymphoid structures and promoted the neogenesis of splenic germinal centers in irradiated mice (84, 144). In the spleen, the Notch2 ligand (delta-like 1, DL1) is present at high concentrations in the small splenic veins which is considered a key activator of MZB development (82, 84).

## 6 Notch signaling pathway is involved in recovery of B cells and HSPCs under irradiation exposure

HSC is a type of cells with self-renewal and differentiation potential in the hematopoietic system (145). It has been shown that exposure to IR doses (>1 Gy) within a short period of time can cause acute radiation sickness, with myeloid acute radiation sickness being the most serious (146). IR inhibits the self-renewal of HSCs and induces the senescence of HSCs mediated by an abnormal increase ROS production, which leads to premature senescence and dysfunction of HSCs (147). In addition, DNA damage induced by IR results in abnormal proliferation and differentiation of HSCs (147, 148), leading to hematopoietic-related diseases such as acute myeloid leukemia (149). Accidental or intentional exposure to moderate to high doses of IR leads to not only acute myelosuppression, but also long-term residual hematopoietic damage manifested as defective HSC self-renewal (150). Correspondingly, it has been recently reported that mice exposed to different doses of IR (2, 4, 6 Gy) within 1 month after exposure had a decrease in the total number of HSPCs and a decrease in the ability of colony formation *in vitro (* 55, 150). Recent findings proved that both endothelial cells and osteoblasts express Notch ligands and promote *ex-vivo* HSPCs maintenance, suggesting that direct ligand or receptor interactions are a key component of the HSPCs ecological niche. In addition, conditional *Notch1* deletion in BM endothelial cells results in reduced HSPCs after irradiation. These data proved for the importance of Notch signaling in maintaining HSPCs in the BM (81, 151).

HSPCs are found in the BM which is the ultimate source of all blood cell lineages (152). Although most hematopoietic lineages develop in the BM, B cell is unique in that it must complete its maturation in peripheral immune organs (153). Furthermore, recent studies have unveiled that Notch signaling can regulate HSC embryonic development, maintenance of “sternness”, and *in vitro* expansion (152, 154). Notch signaling is not only involved in the maintenance of hematopoietic homeostasis (155), but also regulates the development of HSC and B lymphocytes. Endothelial cells express various Notch receptors and ligands to regulate hematopoietic reconstruction in the absence of homeostasis (156, 157). Relevant studies have found that high purity novel Notch ligand heavy histone delta-like receptor 1 (D1R) has the biological effect of targeting anchored endothelial cells and activating the Notch signaling pathway (158). When the BM is acutely or chronically damaged by ionizing radiation, its long-term hematopoietic reconstruction ability is impaired (57). Recombinant protein D1R has the ability to exogenously activate the Notch signaling pathway, a classical pathway of hematopoietic cells within the hematopoietic niche (158, 159). D1R promotes the reconstitution of HSPCs in radiation-damaged mice, endogenously expands hematopoietic cell populations, and contributes to the spectral remodeling of the lymphocyte cells, thus improving the immunity of the body (158, 159).

The BM is the main tissue that produces HSPCs and carries some of the transition from stem cells to differentiated cells, including precursor cells for the different stages of B cells development (160). The stem cells or precursor B cells in the BM are highly susceptible to IR resulting in a dramatic decrease in peripheral B lymphocytes. The hematopoietic system has a strong repair and regenerative capacity. The feature compensates for the decrease in stem cells and lymphoid precursor cells through the activation of the Notch signaling pathway to reestablish the hematopoiesis and maintain homeostatic balance of hematopoiesis *in vivo (* 21, 55, 161). It has been demonstrated that the potential role of Notch in regulating the self-renewal of HSPCs and in determining B cells fates (162). Indeed, a commitment of HSPCs into the B lineage needs to inhibit the Notch1 signal (163). For instance, when Pro-B cells undergo maturation in the BM, bone marrow stromal cells secrete the cytokine CXCL12, which effectively suppresses the expression of Notch ligands (144). With the Pro-B cells continuing to develop, Notch signaling plays an increasingly important role in subsequent developmental processes (134). The differentiation of HSPCs into the B-cell lineage is influenced by distinct Notch ligands and receptors, each playing specific roles (162, 164, 165). For example, Delta-like ligands-1 (Delta 1), as the important Notch2 ligand, induces immature B cells homing to the spleen, where Notch2 activation DLL1-mediated induces immature B2 cells to differentiate into MZB (144). However, the lower densities of Delta 1 in BM is inhibited B lineage development because the induced Notch signaling was not sufficient (162). Relevant researchers found that early B lineage was strongly inhibited in the Delta 1 transgenic NOG mice (NOG-D1-Tg) which have been irradiated 2.5 Gy and transplanted HSC (166). Interestingly, the researcher also showed decreased numbers of B cells in NOG-D1-Tg mice, a similar differentiation rate in B-cell subsets was observed for both NOG-D1-Tg and non-Tg mice (166). This implies that irradiation, in the presence of the Notch signaling ligand Delta, reduced the number of early B cells in the BM, but did not affect the differentiation capacity of B cells. Based on the above studies, we speculate that the depletion effect of radiation on early B cells in BM may be related to the insufficient number of Delta 1 ligands and the silencing of Notch signaling in early B cells. With the continuous differentiation of B cell lineages (134) and the activation of Notch signaling (144, 162), subsequent developing B cells become increasingly resistant to radiation, such as plasma cells (30).

## 7 Prospect and conclusion

Most of the studies on the effects of radiation on the immune system have focused on HSPCs and T cells, but little is known about the influences of radiation on the development and differentiation of B cells. B cells are a specialized class of antigen-presenting cells that produces antibodies to mediate humoral immune responses and activate a large number of cytokines involved in immune regulation, inflammatory responses, and hematopoiesis. B cells are one of the most radiosensitive cells in mammalian cells (4, 23–25)while the mechanisms involved in irradiation-induced B cells damage are still unknown. Notch is an evolutionarily conserved intercellular signaling pathway that regulates cellular differentiation and function at different developmental stages in the spleen, BM, thymus, etc. Interestingly, The Notch pathway has an important role in inducing the development of Pro-B cells to mature B cells during hemopoietic and immune system.

Our present review provides insight into B cells injury from IR and how Notch signaling activates progenitors and precursor B cells to initiate proliferation and differentiation by regulating transcription factors, such as *EBF* and *Pax5*, to replenish damaged B cells in a timely manner. Given that previous research, it is conceivable that Notch regulates B cells to perform non-homologous end-joining for repairing damaged DNA. It is worthwhile to further study that effector B cells (plasma cells) are highly resistant to radiation, which may provide a new idea for radiation therapy of B cells malignancy.

## Author contributions

XS: Writing – original draft. JW: Writing – review & editing. HZ: Writing – review & editing. LS: Funding acquisition, Validation, Writing – review & editing.
